# Leukocyte Telomere Length Correlates with Extended Female Fertility

**DOI:** 10.3390/cells11030513

**Published:** 2022-02-02

**Authors:** Jennia Michaeli, Riham Smoom, Noa Serruya, Hosniyah El Ayoubi, Keren Rotshenker-Olshinka, Naama Srebnik, Ofir Michaeli, Talia Eldar-Geva, Yehuda Tzfati

**Affiliations:** 1 Department of Obstetrics and Gynecology, Shaare Zedek Medical Center Affiliated with the Hebrew University School of Medicine, Jerusalem 9103102, Israel; kerenlogic@gmail.com (K.R.-O.); srebnik@gmail.com (N.S.); gevat@szmc.org.il (T.E.-G.); 2 Department of Genetics, The Silberman Institute of Life Sciences, The Hebrew University of Jerusalem, Givat Ram, Jerusalem 91904, Israel; riham.samman@mail.huji.ac.il (R.S.); noaserruya96@gmail.com (N.S.); hosniyah.el@mail.huji.ac.il (H.E.A.); ofir@tty.co.il (O.M.)

**Keywords:** telomeres, longevity, female fertility, reproductive aging

## Abstract

Current social trends of delayed reproduction to the fourth and fifth decade of life call for a better understanding of reproductive aging. Demographic studies correlated late reproduction with general health and longevity. Telomeres, the protective ends of eukaryotic chromosomes, were implicated in various aging-associated pathologies and longevity. To examine whether telomeres are also associated with reproductive aging, we measured by Southern analysis the terminal restriction fragments (TRF) in leukocytes of women delivering a healthy infant following a spontaneous pregnancy at 43–48 years of age. We compared them to age-matched previously fertile women who failed to conceive above age 41. The average TRF length in the extended fertility group (9350 bp) was significantly longer than in the normal fertility group (8850 bp; *p*-value = 0.03). Strikingly, excluding women with nine or more children increased the difference between the groups to over 1000 bp (9920 and 8880 bp; *p*-value = 0.0009). Nevertheless, we observed no apparent effects of pregnancy, delivery, or parity on telomere length. We propose that longer leukocyte telomere length reflects higher oocyte quality, which can compensate for other limiting physiological and behavioral factors and enable successful reproduction. Leukocyte telomere length should be further explored as a novel biomarker of oocyte quality for assessing reproductive potential and integrating family planning with demanding women’s careers.

## 1. Introduction 

In the modern era, as part of the social trends of delayed reproduction, an increasing number of women prefer to postpone childbearing to the fourth and fifth decade of their lives. This narrows down the window of opportunities, since natural conceptions, and especially the ability to deliver a genetically healthy child, decline rapidly and become rare during the fifth decade of life [1]. These social trends, given the natural fertility limitation, pose an important diagnostic and management challenge [2,3]. The main reason for the decrease in pregnancy rates and the increase in miscarriages and genetically abnormal conceptions is the decline in oocyte quantity and quality associated with advanced maternal age [4]. We possess several surrogate markers of oocyte quantity, often referred to as the ovarian reserve [5]. However, to date, there is no reliable biomarker of oocyte quality that can aid in the assessment and treatment of age-related infertility. Demographic studies among different ethnic groups and different epochs positively correlated late female reproduction with signs of general health and longevity [6,7,8,9,10]. These studies suggest that extended fertility and delayed aging have a common genetic background.

Telomeres are highly conserved nucleoprotein complexes composed of tandem six nucleotide DNA repeats and associated proteins [11]. They protect the ends of eukaryotic chromosomes from being recognized as double-strand breaks by the DNA damage response machinery. Throughout life, telomeres in somatic tissues gradually shorten with each cell division and age. On the cellular level, telomere attrition can ultimately lead to cell cycle arrest and cell senescence. Thus, short telomeres were suggested to cause aging-related pathologies, and long telomeres are associated with longevity, as well as increased risk of various cancers [11,12]. While telomeres in the sperm were shown to elongate with age, this is not the case in the oocytes [13]. It has been suggested that short telomeres in the oocytes are associated with reduced success rates of in vitro fertilization (IVF) [14,15]. However, the association of age-related telomere shortening with normal fertility has not been reported. 

We hypothesized that telomere length links extended natural female fertility and longevity. To examine this hypothesis, we measured leukocyte telomere length in women displaying extended fertility compared to women with normal fertility. Our findings indicate that leukocyte telomere length correlates with female fertility at an advanced age, suggesting an important role for telomeres in reproductive aging. We propose that telomere length should be further explored to understand the mechanistic role of telomeres in reproductive aging, and as a useful tool to guide treatment options and assist in family planning and fertility preservation choices. 

## 2. Materials and Methods

### 2.1. Study Design and Participants

This study is a retrospective case–control study conducted in 2018–2020. The main study group, termed extended fertility (EF), consisted of women delivering a healthy infant following a spontaneous pregnancy at 43 to 48 years of age. The control group, termed normal fertility (NF), consisted of age-matched, previously fertile, and currently with regular menses, parous women who did not use contraceptives, but failed to conceive and deliver a healthy child after the age of 41 years despite attempts to conceive. Both study groups belong to an orthodox population with religious motivation to continue baring children as long as nature permits. We measured leukocyte telomere length in the two groups. In order to assess the immediate effect of pregnancy and delivery in the EF group, one blood sample was collected within 48 h of delivery, and an additional sample at least 5 months postpartum. A single sample was collected in the NF group when recruited for the study. To test the cumulative effect of parity on telomere length, we compared leukocyte telomere length in primiparous (first delivery) women age 30 to 35 years to grand-multiparous (6 or more deliveries) women of the same age. All children born to participants in the study were considered healthy if no malformations were detected after birth and no known genetic syndromes were identified (e.g., Down’s or Turner syndromes). 

### 2.2. Leukocyte Genomic DNA Preparation

Whole blood samples were collected in EDTA tubes. Erythrocytes were lysed using an erythrocytes lysis buffer [155 mM NH_4_Cl, 12 mM NaHCO_3_, 0.1 mM EDTA], and white blood cells were collected. Genomic DNA was extracted through the standard proteinase K and phenol-chloroform extraction method.

### 2.3. Telomere Length Measurements

Mean telomere length was measured by Southern analysis of telomere terminal restriction fragments (TRF), as previously described [16]. Genomic DNA samples (5 µg) digested by HinfI were electrophoresed in 0.7 % agarose, 1 × TBE, for 1800 V × H, transferred to a Hybond N^+^ membrane (GE Healthcare) by a vacuum blotter (model 785, Bio-Rad Inc.), hybridized to a 5′ ^32^P-labeled telomeric probe, (AACCCT)_3_, and exposed to Typhoon FLA 9500 PhosphorImager (GE Healthcare Inc., Hercules, CA, USA). Raw data were analyzed by TeloTool software (corrected mode), specifically designed to obtain average TRF length for each sample [17]. Sample measurements were reproducibly repeated several times in different gels and averaged.

### 2.4. Statistical Analysis

For categorical variables, statistics are provided as numbers and percentages. For continuous variables, statistics are described by mean values with standard deviations. The effect of categorical variables on continuous measurements was tested by paired and unpaired two-sided Student’s t-test. A *p*-value < 0.05 was considered statistically significant. Statistics were computed and presented by Microsoft Excel 2016 (with the Data-analysis add-in) and GraphPad Prism 8. 

## 3. Results

### 3.1. Women with Extended Fertility Have Longer Telomeres 

**Figure 1:**
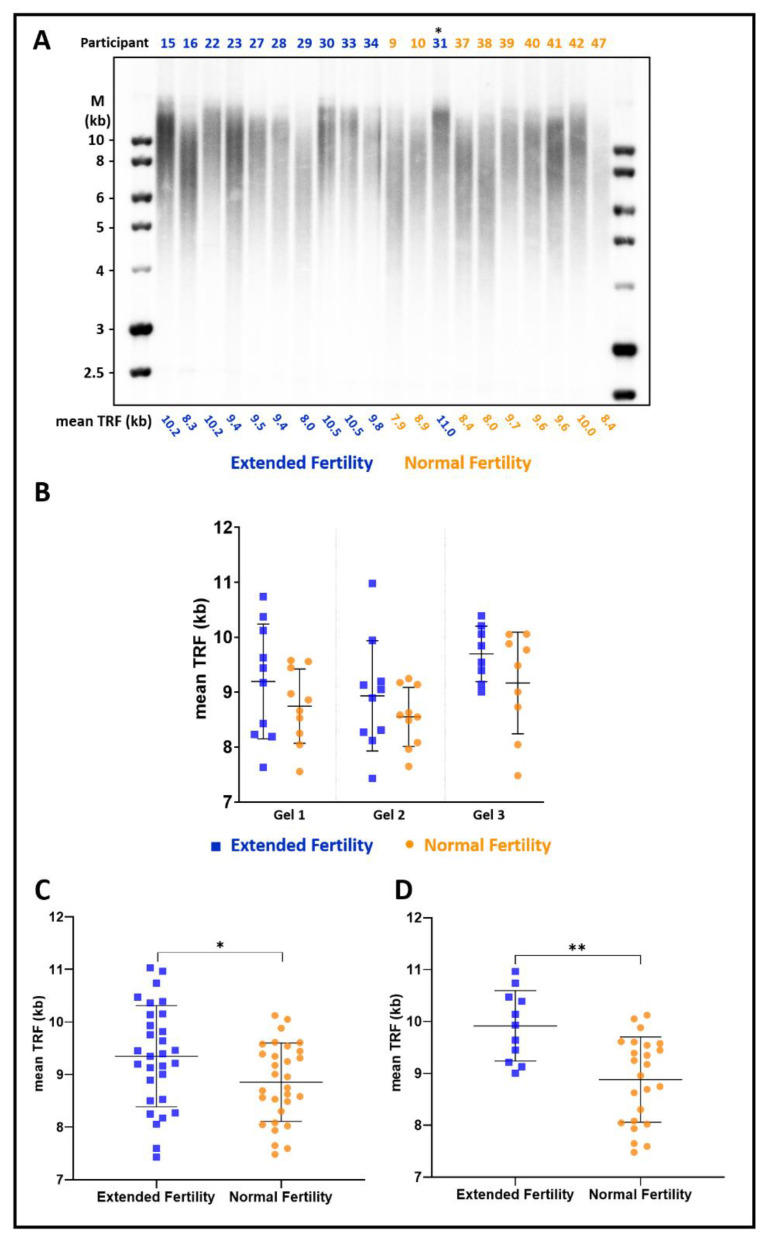
Women with extended fertility have longer telomeres. (**A**) A representative image of a Southern blot hybridized with a telomeric probe. Blood samples were collected from the Extended Fertility (EF) group participants (blue) within 48 h after delivery and the Normal Fertility (NF) group participants at recruitment (orange), and their leukocyte telomere length was analyzed as described under ‘Materials and Methods’. The mean telomere terminal restriction fragment (TRF) length, as calculated by TeloTool, is depicted below each lane. (**B**) Mean TRF in the NF and EF groups measured in three separate non-redundant gels shown in (**A**) and in the Supplementary Figure S1. Note that in (**A**), one of the women sampled as NF (indicated by an asterisk) later conceived and delivered a healthy child, thus reclassified as EF. (**C**) Mean TRF length reproducibly measured in several different gels for each participant, averaged, and presented for all the participants of the EF versus NF groups. *p*-value = 0.03 (*). Indicated are average and SD. (**D**) Mean telomere length is presented for a subgroup of participants with up to eight children in the EF and NF groups. *p*-value = 0.0009 (**). Indicated are average and SD. The graph for the subgroup of women with 9 or more children is shown in Supplementary Figure S3.

To examine the association of telomeres with extended fertility in women, we measured telomere length in sixty women 43–48 years old: Thirty women who naturally conceived and delivered a healthy baby in the extended fertility (EF) group and thirty women who failed to conceive above the age of 41 in the normal fertility (NF) group. The mean age in the EF group was 44.2 years and in the NF group 44.9 years (Table 1). Mean parity was 9.4 in the EF group and 5.7 in the NF group. All women are of Caucasian ethnicity, leading a similar lifestyle and equal socio-economic status. Additional demographic characteristics were similar between the groups, as summarized in Table 1. Mean telomere terminal restriction fragments (TRF) length was measured in three non-redundant separate gels, each including randomly assigned non-redundant EF and NF samples that were collected 48 h after delivery for the EF group and at recruitment for the NF group (representative gels are shown in Figure 1A and Supplementary Figure S1A,B). In each independent gel, the average mean TRF length in the EF group was significantly longer than in the NF group (*p* < 0.05; Figure 1B). Additional gels, with different combinations of randomly assigned samples, were run to validate the results and increase the accuracy of the mean TRF length measured for each participant (an example is shown in Supplementary Figure S1C and all the results for each sample are summarized in Table S1). Averaging the data from all the gels yielded an average mean TRF length in the EF group of 9350 ± 950 bp, significantly longer than that of the NF group—8850 ± 740 bp (*p*-value= 0.03; Figure 1C).

### 3.2. The Difference in Telomere Length Is More Pronounced in Women with Up to Eight Children 

When plotting mean TRF length according to parity for the EF group, we noticed a significant difference between women with up to 8 children and women with 9 or more (Supplementary Figure S2). Since the NF group did not show such correlation, we suspected that it did not reflect an effect of pregnancy or delivery on telomere length. Rather, the EF group was a mixture of two sub-populations, and extended fertility was associated with longer telomeres in only one of these sub-populations. Indeed, among the women with up to eight children, the difference in average mean TRF length between EF and NF groups was over 1000 bp (9920 ± 650 vs. 8880 ± 800, *p*-value= 0.0009; Figure 1D). In contrast, among the women with nine or more children, the difference between EF and NF was not significant (Supplementary Figure S3).

### 3.3. Pregnancy, Delivery, and Parity Do Not Affect Telomere Length

**Figure 2:**
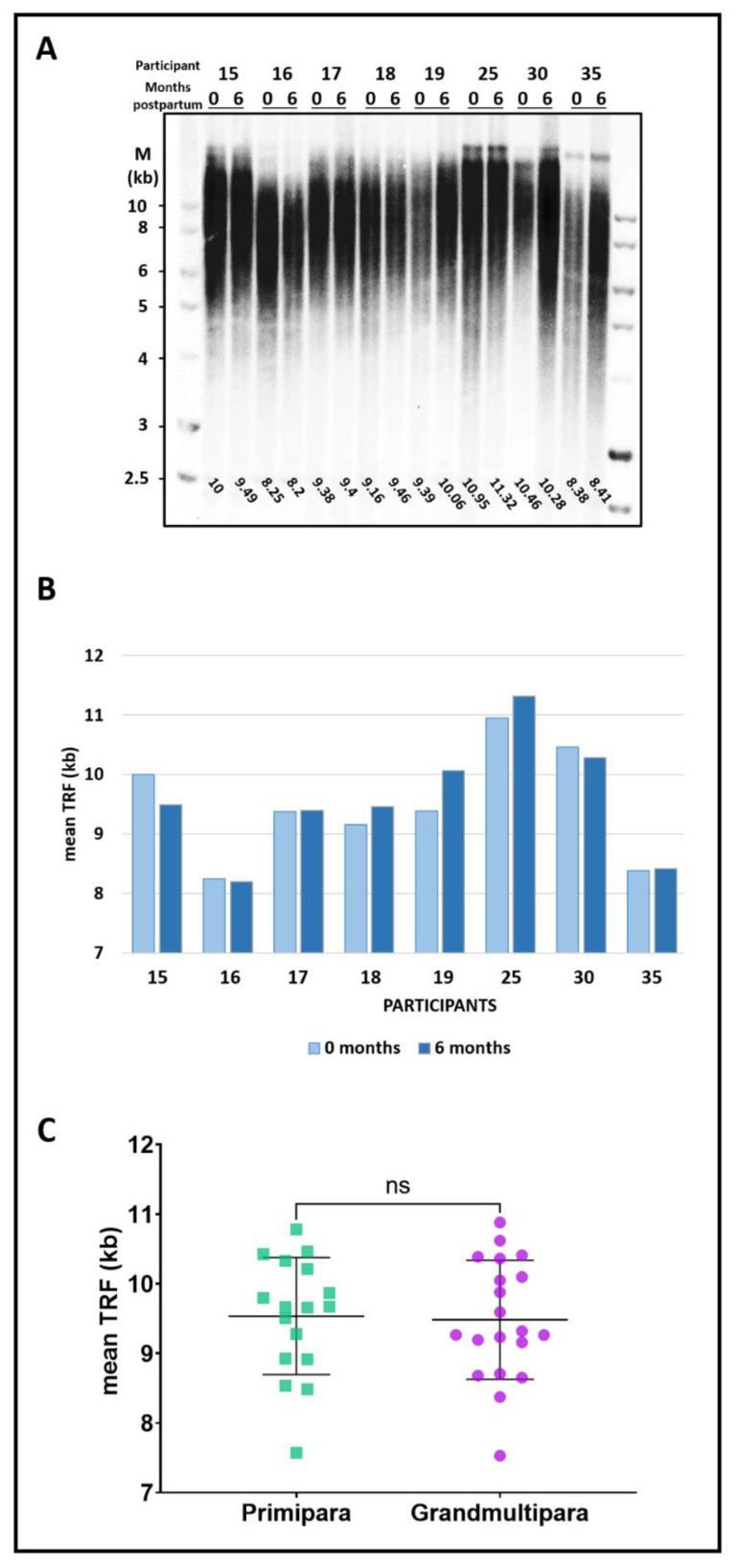
No apparent association of childbearing and childcaring with telomere length. (**A**) Leukocyte telomere length for each of the participants of the EF group was measured within 48 h of delivery (‘0’) and again five to six months later (‘6’). (**B**) Graph showing the mean TRF values for each participant, at delivery (light blue) and six months postpartum (dark blue). *p*-value = ns. (**C**) Graph showing mean TRF length measured in primiparous (first delivery, green) versus grand-multiparous (≥6 deliveries, purple). *p*-value = ns. The Southern images are shown in Supplementary Figure S2.

To further examine the short-term effects of pregnancy and delivery on telomere length, we compared mean TRF length measured in the EF group within 48 h of the delivery to samples obtained from the same participants five to six months later. As shown in Figure 2A,B and Supplementary Figure S4A, there was no significant difference in leukocyte mean TRF length measured for each woman between the two samples (*p*-value = ns; *n* = 11), excluding short-term effects of pregnancy and delivery.

Next, we examined the long-term effects of parity on telomere length by comparing mean TRF length in another group of women between 30 to 35 years of age, in 17 primiparous women (first delivery) to 20 grand-multiparous women (who delivered their 6th to 11th child; see Table 2). The mean age of the participants was 31.5 ± 1.3 and 32.9 ± 1.2 for the primiparous and grand-multiparous groups, respectively. We found no significant difference in telomere length between the groups (9540 ± 820 bp for primiparous versus 9490 ± 840 bp for grand-multiparous women, *p*-value = ns; Figure 2C and representative gels in Supplementary Figure S4B,C).

## 4. Discussion

### 4.1. Women with Unusual Extended Fertility Have Longer Telomeres

Demographic studies described a longer lifespan in women with extended fertility [7,10]. Here, we show that longer telomeres are strongly associated with the rare ability to spontaneously conceive at an advanced age and deliver a healthy child (Figure 1), and thus provide a link between fertility and longevity. The difference in telomere length was particularly pronounced in a subset of women having up to eight children, displaying more than 1000 bp difference in mean TRF length between the EF and NF groups (9920 ± 650 vs. 8880 ± 800; *p*-value = 0.0009; Figure 1D). Women with nine or more children did not display a significant correlation (Supplementary Figure S3). 

### 4.2. Parity and Delivery Do Not Affect Average Telomere Length 

The association of parity with telomere length in the EF group raised two possible explanations. According to one, telomeres shorten with the accumulating number of pregnancies and deliveries or period of caregiving, hindering the initially longer telomere length set point in the EF women having nine or more children. Indeed, some demographic studies reported a possible trade-off between fertility and longevity, suggesting that increased parity is correlated with shorter telomeres and a shorter lifespan [6,18,19]. However, other reports suggest otherwise [8]. Alternatively, our observation could indicate that other factors not associated with telomere length facilitate extended fertility in the group of women with nine or more children. To distinguish between these possibilities, we examined the short-term effects of pregnancy and delivery and the long-term cumulative effect of parity on telomere length (Figure 2 and Supplementary Figure S4). We found no such apparent effects, excluding the former possibility and suggesting that other factors play a major role in facilitating fertility in this group of women having nine or more children. 

### 4.3. Telomere Length as a Biomarker for Oocyte Quality 

While Anti-Müllerian Hormone (AMH) is an accepted measure of oocyte quantity, no biomarkers are known for oocyte quality, which relates to the potential of a fertilized oocyte to develop into a genetically healthy newborn [20]. AMH levels correlate with the response to ovarian stimulation and oocyte yield in artificial reproductive technology cycles [21], but not with its outcome—live birth rate [22]. Lessons from elective fertility preservation through oocyte vitrification have shown that for the same number of vitrified oocytes, the cumulative live birth rate declines considerably with increased maternal age [23]. These data reflect the more critical role of oocyte quality over quantity. While telomere length is highly variable among individuals and there is overlap in the mean TRF length between the EF and NF groups (Figure 1C), all women in the EF group with up to 8 children have a mean TRF length of above 9 kb (Figure 1D). Therefore, we propose that leukocyte telomere length should be further explored as a biomarker for oocyte quality, which would help predict the chances to conceive at advanced maternal age. While it may not be useful as a biomarker for women with nine or more children, our results suggest that its predictive value increases for women with fewer or no children at all and a lower chance of conceiving and completing a successful pregnancy. 

### 4.4. A Working Model for Extended Fertility 

**Figure 3:**
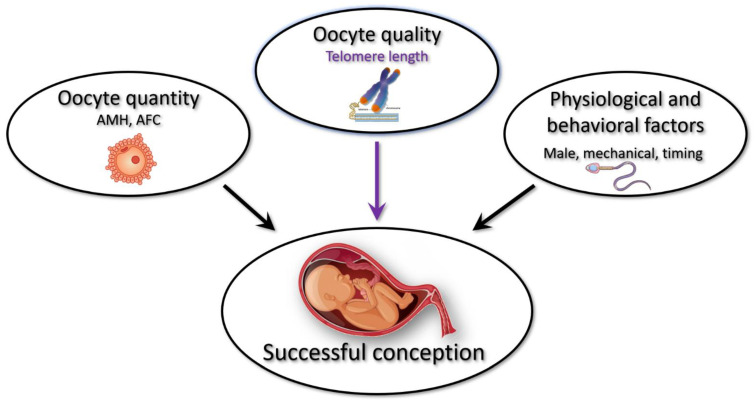
A working model for extended fertility. Female fertility at an advanced age is determined by a combination of factors: the quality of the oocytes (represented by telomere length), the oocyte quantity (represented by AMH levels or Antral Follicle Count), and male and physiological–behavioral characteristics (represented by sperm count, proper timing of intercourse, demonstration of normal female anatomy, etc.). While low AMH levels are permissive to extended fertility, oocyte quality and physiological and behavioral factors may be limiting and compensating for each other.

Conception, pregnancy, and delivery of a healthy child are complex events with a large number of contributing factors, including female, male, and behavioral–environmental inputs, as well as arbitrary chance. Maternal age is known as a primary determinant of female fertility, which is limited by the decline in oocyte quantity and oocyte quality [1]. According to our working model, female fertility at an advanced age is determined by a combination of factors: the quality of the oocytes, the quantity of the oocytes, and male and physiological–behavioral characteristics (Figure 3). We found a strong correlation between telomere length and extended fertility in women with up to eight children. Since these women do not use contraceptives, we speculate that the relatively smaller number of children reflects lower chances for fertilization and conception due to varying physiological or behavioral limiting factors. In this context, higher oocyte quality, correlated with longer telomeres is more critical and can compensate for the lower chance to conceive and enable successful reproduction. On the other hand, in the group of women with nine or more children, physiological or behavioral factors are not limiting (thus the higher number of children), providing more fertilization opportunities, compensating for the aging-reduced oocyte quality and maintaining a reasonable chance for successful conception. While our working model is hypothetical at this stage, it sets the framework for further research of various factors affecting female fertility, and specifically the association of telomeres with female reproductive aging.

### 4.5. Do Telomeres Play a Direct Role in Extended Fertility? 

Healthy telomeres have a cardinal role in gametogenesis and the proper function of the germ cells throughout reproductive life [24]. Telomeres participate in early meiosis by linking chromosomes to the nuclear envelope, facilitating bouquet and homologous synapsis formation, and allowing the essential meiotic recombination events to take place [25]. Accumulating damage to telomeres of germ cells leads to errors in meiosis and may result in aneuploid gametes. Indeed, female reproductive aging has been attributed to the increasing number of genetically abnormal gametes due to maternal nondisjunction [2,4,26,27,28,29]. Additionally, in the murine model, it was found that telomere shortening and malfunction, associated with decreased expression of the sirtuin SIRT6, is an important pathway connecting maternal aging with the decline in oocyte integrity and the increased risk for defects during early embryonic development [26]. Furthermore, it has been suggested that telomere shortening in the oocytes due to oxidative damage is associated with reduced success rates of IVF [14,15]. While it is difficult to obtain and measure telomere length in an individual or a small number of oocytes, blood is readily available for research and diagnosis. Yet, a valid concern is how well can leukocyte telomere length represent non-dividing oocyte. It was proposed that telomere length is highly correlated among different tissues of an individual [30,31]. Indeed, leukocyte telomere length is commonly used as a biomarker, representing telomere length in other cell types and indicating physiologic and pathologic states in various tissues [32]. Thus, it is reasonable to assume that leukocyte telomere length also reflects the telomere state in the oocytes. While we cannot rule out that longer leukocyte telomeres merely reflect a younger physiological age, we favor the possibility that sufficiently long telomeres in the oocytes increase the chances for successful completion of the first and second meiosis with fewer chromosomal aberrations and less aneuploidy. This way, healthy telomeres could play a direct role in facilitating extended fertility.

## 5. Conclusions

Based on our findings, we propose that leukocyte telomere length is associated with women’s fertility at advanced ages and possibly plays an important role in reproductive aging. To date, there are no surrogate markers for pregestational oocyte quality, and average telomere length can serve as a biomarker for oocyte quality and help predict the chances to conceive at advanced maternal age. Such a biomarker is essential to facilitate personalized fertility counseling and patient-tailored infertility treatments. Finally, the telomere link between longevity and extended fertility described here provides an important insight into a mechanism contributing to ovarian aging and subfertility, which will be explored further in future research.

## Figures and Tables

**Table 1:** Demographic characteristics of EF and NF study groups.

	Case (*n* = 30)	Control (*n* = 30)
Age (years), mean ± SD	44.23 ± 1.40	44.9 ± 1.35
Gravida, mean ± SD	11.63 ± 5.03	7.5 ± 4.09
Para, mean ± SD	9.37 ± 3.83	5.7 ± 3.27
Miscarriages, mean ± SD	2.27 ± 1.93	1.77 ± 1.84
Living children, mean ± SD	9.4 ± 3.89	5.83 ± 3.25
Menarche (years), mean ± SD	13.14 ± 1.68	13.28 ± 1.41
Mother’s age at last child (years), mean ± SD	37.19 ± 5.56	36.93 ± 5.58
Sister’s age at last child (years), mean ± SD *	41.18 ± 2.32	38.87 ± 4.14

* For case study group *n* = 17, control study group *n* = 15, as some participants do not have sisters or sisters that are still in childbearing ages.

**Table 2:** Demographic characteristics of primipara and grandmultipara study groups.

	Primipara	Grandmultipara
	(*n* = 17)	(*n* = 20)
Age (years), mean ± SD	31.47 ± 1.33	32.95 ± 1.24
Gravida, mean ± SD	1.29 ± 0.75	8.30 ± 1.55
Para, mean ± SD	1	7.45 ± 1.32
Miscarriages, mean ± SD	0.29 ± 0.75	0.85 ± 1.11
Living children, mean ± SD	1	7.40 ± 1.36
Menarche (years), mean ± SD	13.71 ± 1.52	12.90 ± 0.85
Mother’s age at last child (years), mean ± SD	34.69 ± 5.11	39.15 ± 4.38

## Data Availability

All deidentified participants’ data collected for the study, study protocols, statistical analysis, and results will be available upon request from the corresponding authors and a signed data access agreement.

## Supplementary Materials

The following are available online at https://www.mdpi.com/article/10.3390/cells11030513/s1, Figure S1: Additional Southern blot images of participants in the study groups, Figure S2: Correlation between parity and mean telomere length in the EF group, Figure S3: EF and NF participants with nine or more children do not display a significant difference in telomere length, Figure S4: Additional Southern blot images of participants in the study groups, and Table S1: mean TRF per study participant.
