# Supplementary material for: Leukocyte Telomere Length Correlates with Extended Female Fertility
Source: Cells. 2022 Feb 2;11(3):513. doi: 10.3390/cells11030513 (PMC8834216; doi:10.3390/cells11030513)
Supplement: Supplementary file 1 [file cells-11-00513-s001.zip › cells-1538658-Supplementary material 22.2.8/cells-1538658-Supplementary material cells final version.pdf]

Leukocyte Telomere Length Correlates with Extended Female Fertility

Jennia Michaeli[a,b,1], Riham Smoom[b], Noa Serruya[b], Hosniyah El Ayoubi[b], Keren Rotshenker-Olshinka[a], Naama Srebnik[a], Ofir Michaeli[b], Talia Eldar-Geva[a], Yehuda Tzfati[b*]

[a]Department of Obstetrics and Gynecology, Shaare Zedek Medical Center Affiliated with the Hebrew University School of Medicine, Jerusalem, 9103102, Israel

[b]Department of Genetics, The Silberman Institute of Life Sciences, Safra Campus, The Hebrew University of Jerusalem, Jerusalem, 91904, Israel.

[1]Present address: Mount Sinai Fertility, Sinai Health System, 250 Dundas St. West, Suite 700, Toronto, Ontario M5T 2Z5 Canada

Supplementary figures

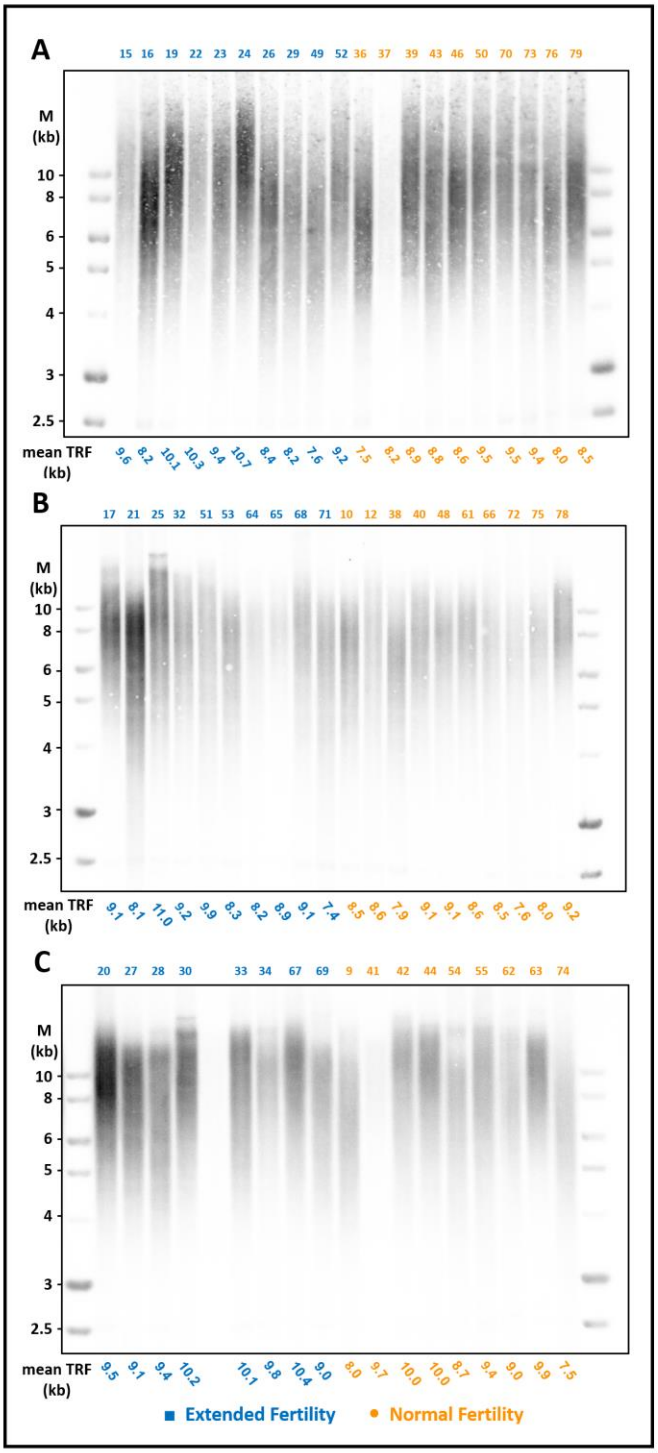

Figure S1. Additional Southern blot images of participants in the study groups

Blood samples were collected from the Extended Fertility (EF) group participants (blue) within 48 hours after delivery and the Normal Fertility (NF) group participants at recruitment (orange), and their leukocyte telomere length was analyzed as described under 'Methods'. The mean telomeric restriction fragment (TRF) length, as calculated by *TeloTool* is depicted below each lane. The gels in (A,B), as well as in Figure 1A of the main text, were designed to include randomly assigned non-redundant participants p-value<0·05 in each gel. (C) An additional representative gel with duplicated samples.

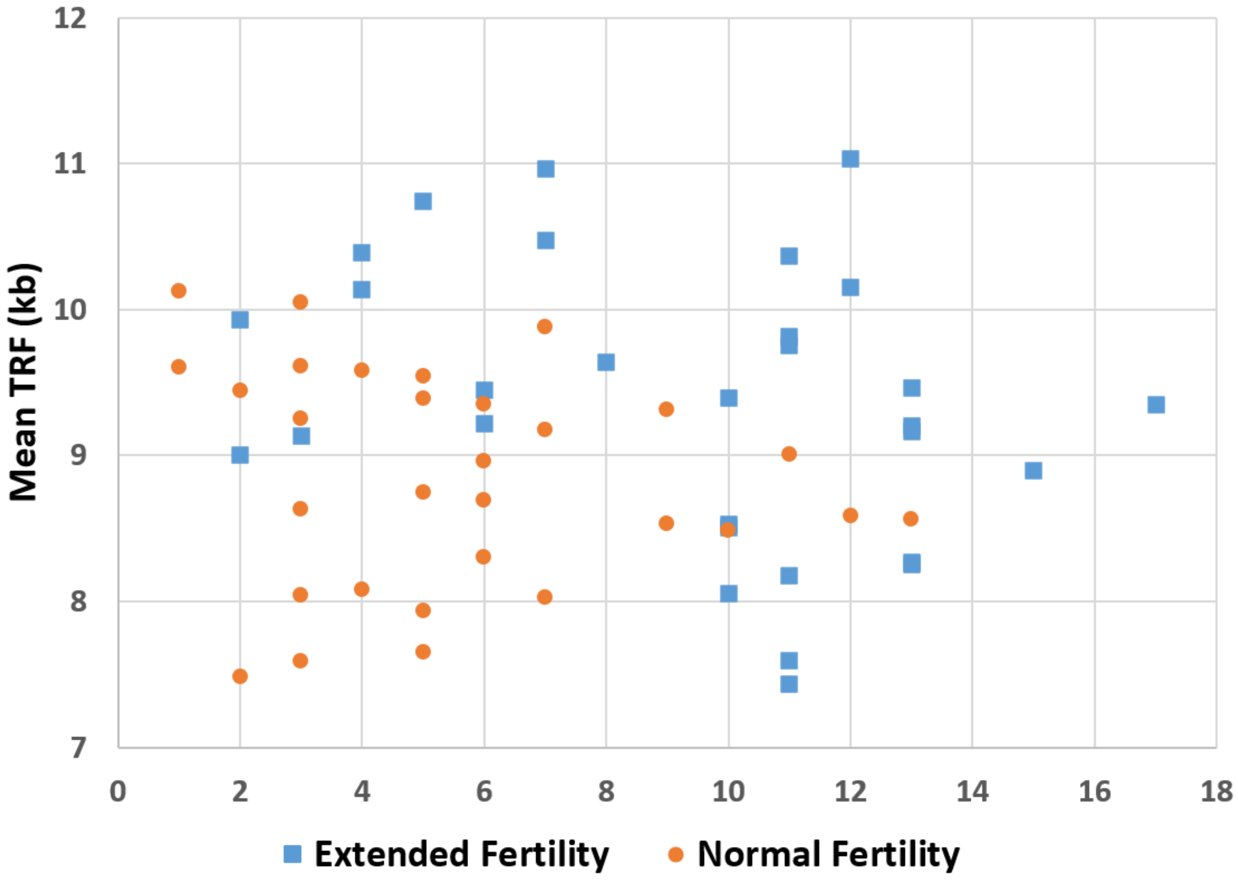

Figure S2. Correlation between parity and mean telomere length in the EF group
Graph showing the mean TRF length per number of children for each participant of the EF and NF groups.

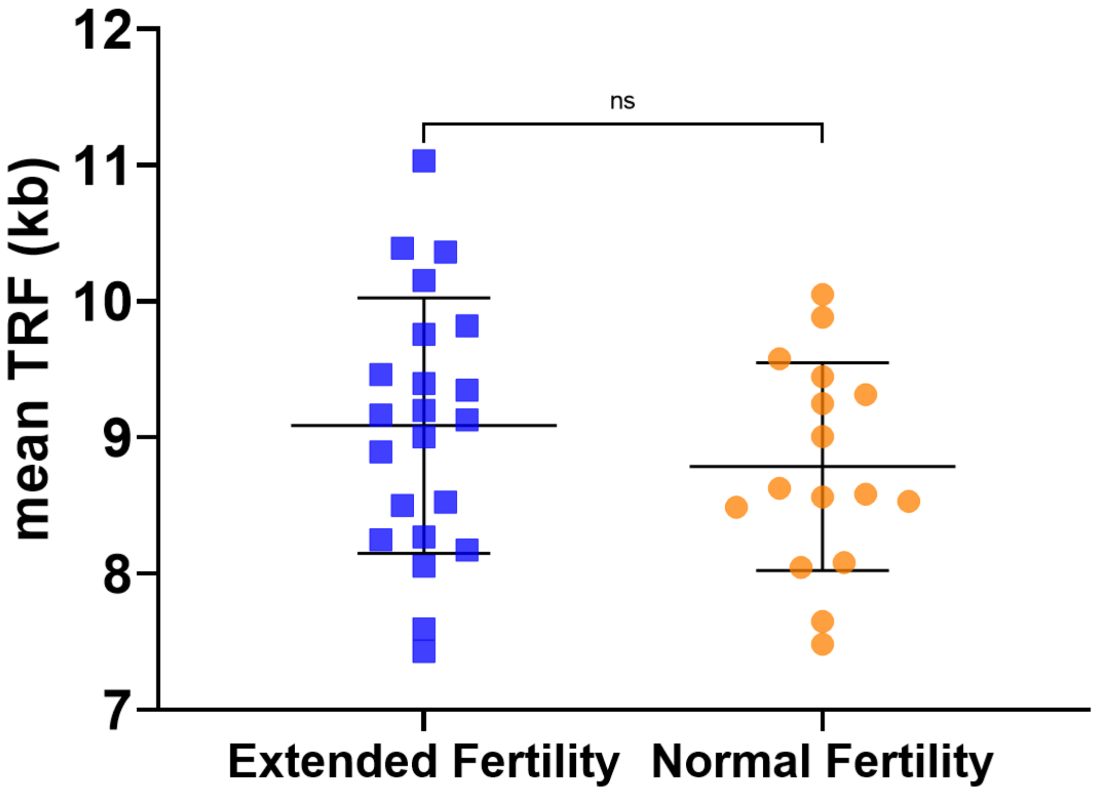

Figure S3. EF and NF participants with nine or more children do not display a significant difference in telomere length

Mean leukocyte telomere length is presented for a subgroup of participants with nine or more children in the EF (blue □) *versus* NF (orange ○) groups. Indicated are average and SD. p-value= ns.

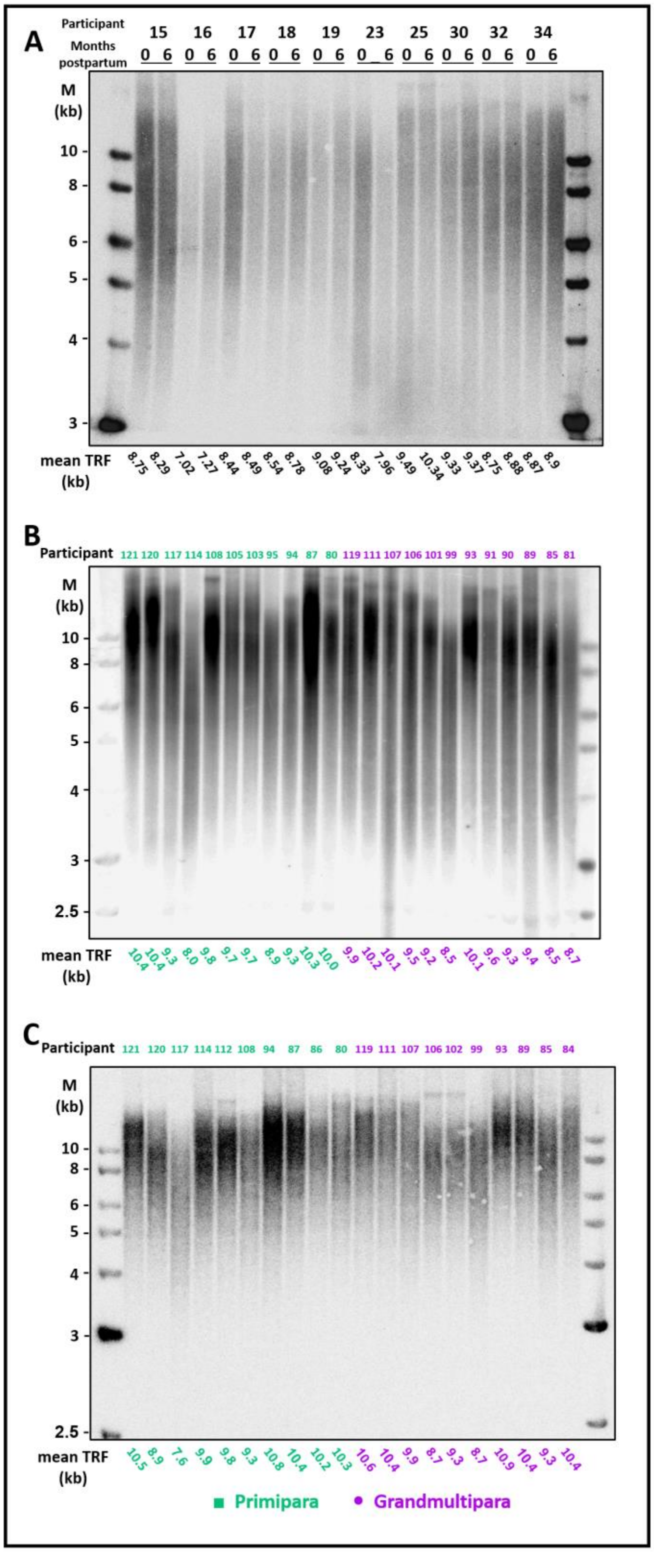

Figure S4. Additional Southern blot images of participants in the study groups

(A) Additional gel showing samples collected from Extended Fertility (EF) group participants at delivery (0) and five to six months later (6) p-value=ns. (B,C) Mean TRF length was measured in primiparous (first delivery, green) *versus* grandmultiparous woman (≥6 deliveries, purple), as described under Figure 1A and 'Methods'. Two representative gels are shown. p-value=ns.
