# Supplementary figures and images for: Leukocyte Telomere Length Correlates with Extended Female Fertility
Source: Cells. 2022 Feb 2;11(3):513. doi: 10.3390/cells11030513 (PMC8834216; doi:10.3390/cells11030513)

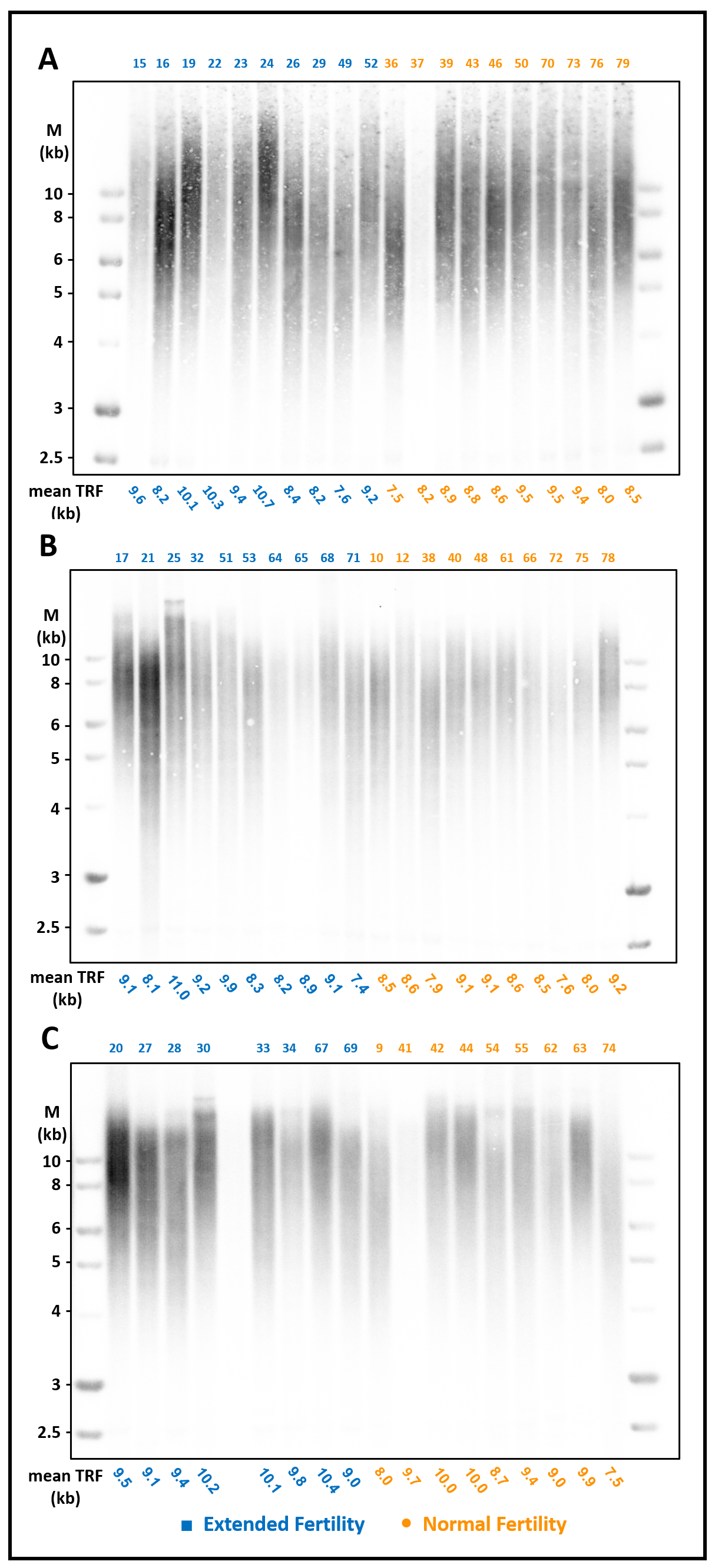

Supplement: Supplementary file 1 [file cells-11-00513-s001.zip › cells-1538658-Supplementary material 22.2.8/Figure S1.png]

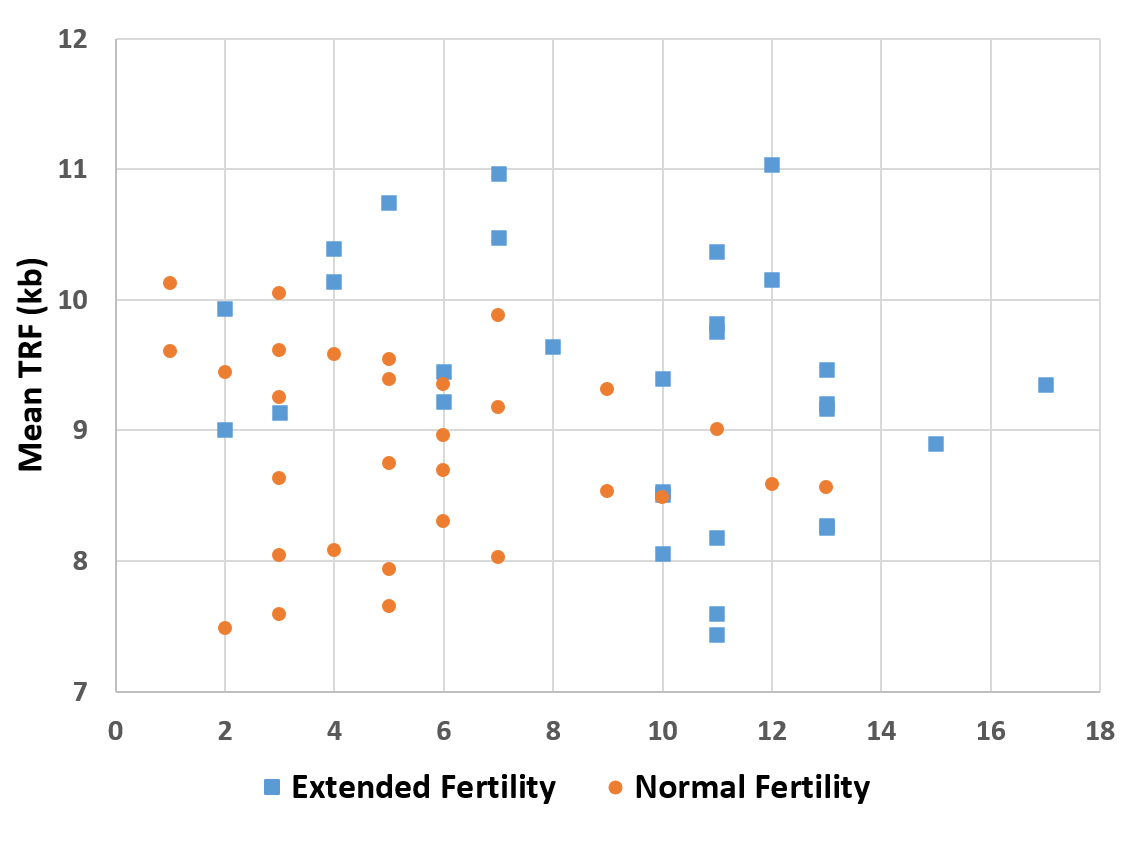

Supplement: Supplementary file 1 [file cells-11-00513-s001.zip › cells-1538658-Supplementary material 22.2.8/Figure S2.png]

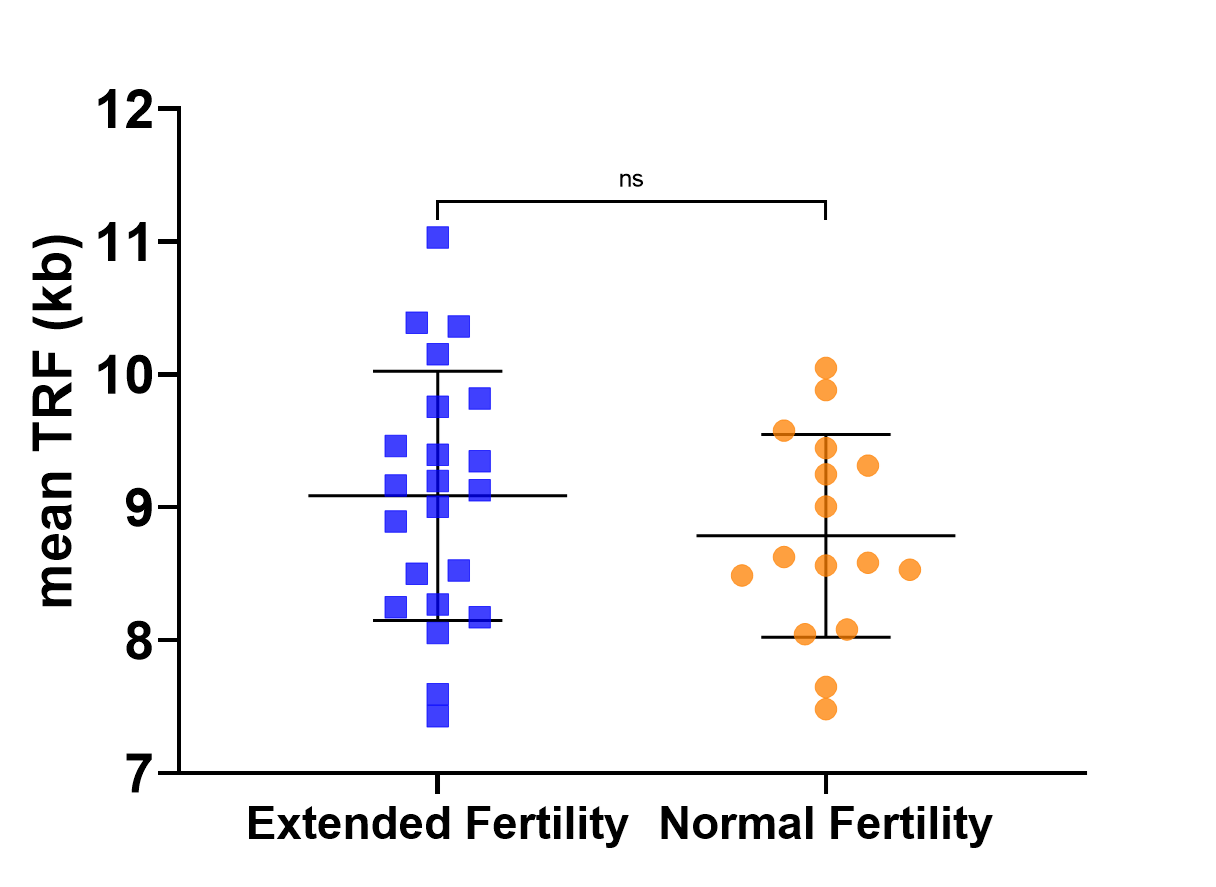

Supplement: Supplementary file 1 [file cells-11-00513-s001.zip › cells-1538658-Supplementary material 22.2.8/Figure S3.png]

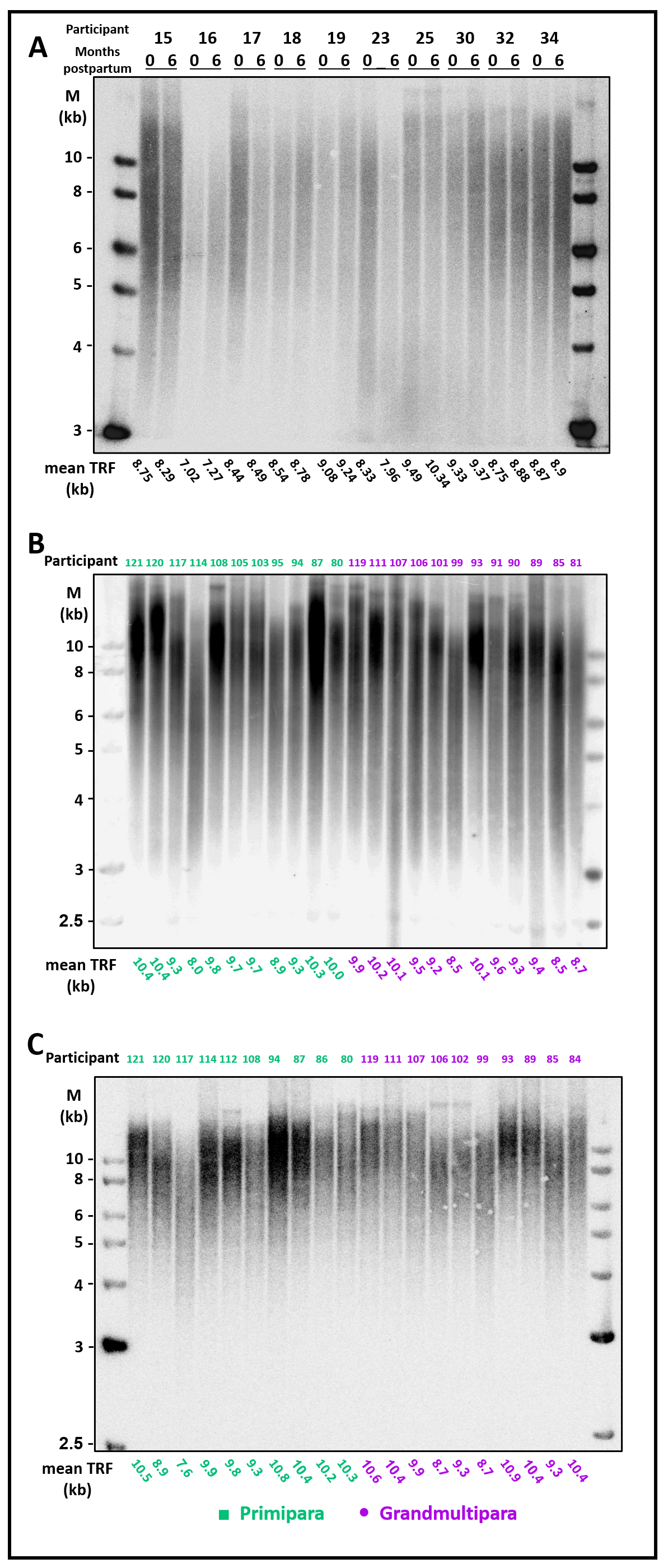

Supplement: Supplementary file 1 [file cells-11-00513-s001.zip › cells-1538658-Supplementary material 22.2.8/Figure S4.png]
